# Exploring the Potential Mechanism of *Smilax Glabra* Roxb in Periodontitis Through Network Pharmacology and Molecular Docking

**DOI:** 10.2174/0113816128391490250729102829

**Published:** 2025-08-08

**Authors:** Jinjia Hong, Peilun Ma, Yuan Zhang, Na Li, Pengfei Zhang, Chunrui Tian, Yukang Cao, Xing Wang

**Affiliations:** 1 Shanxi Medical University School and Hospital of Stomatology, Taiyuan, 030001, China;; 2 Shanxi Province Key Laboratory of Oral Diseases Prevention and New Materials, Taiyuan, 030001, China

**Keywords:** Smilax glabra Roxb, periodontitis, network pharmacology, molecular docking, IL6, KEGG

## Abstract

**Introduction:**

This study aims to explore the potential mechanisms of Smilax Glabra Roxb (SGR) in the treatment of periodontitis using network pharmacology and molecular docking.

**Methods:**

The active components and targets of SGR were identified using the TCMSP, STITCH, and SwissTargetPrediction databases, while periodontitis-related targets were retrieved from GeneCards, TTD, and OMIM. Overlapping targets were subjected to Protein-Protein Interaction (PPI) analysis *via* the STRING platform, followed by network analysis using Cytoscape software and the MCODE plugin to identify key protein targets. Gene Ontology (GO) and Kyoto Encyclopedia of Genes and Genomes (KEGG) pathway enrichment analyses were conducted through the DAVID database to determine relevant biological processes and pathways. Finally, molecular docking was performed to assess the binding affinity between the key active components of SGR and critical target and pathway proteins.

**Results:**

A total of 15 active components and 527 potential targets of SGR were identified, along with 367 targets related to periodontitis. The 75 overlapping targets were considered potential therapeutic targets. Key genes, such as IL6, IL1Β, and TNF, were identified through Cytoscape analysis. KEGG enrichment analysis indicated that the overlapping targets are primarily involved in inflammatory and metabolic pathways, including the AGE-RAGE signaling pathway, the lipid and atherosclerosis pathway, and the TNF signaling pathway. Molecular docking results showed that the key active components can stably bind to the critical targets and pathway proteins.

**Discussion:**

SGR may have particular advantages in treating periodontitis in patients with systemic diseases, such as diabetes and cardiovascular conditions, but further research is needed to validate its clinical efficacy and safety.

**Conclusion:**

This study highlights the therapeutic potential of SGR in the treatment of periodontitis and elucidates its possible molecular mechanisms, offering new insights and targets for periodontitis therapy.

## 1 INTRODUCTION

Periodontitis is a common, chronic inflammatory disease that primarily affects the periodontal supporting tissues, including the gums, periodontal ligaments, and alveolar bone [1-3]. It is characterized by the progressive destruction of periodontal tissues, which may eventually lead to tooth mobility and even loss [4, 5]. Periodontitis not only affects oral health but is also closely associated with various systemic diseases, such as diabetes, cardiovascular diseases, preterm birth, and other inflammatory conditions [6-8]. Studies suggest that chronic inflammation in patients with periodontitis may exacerbate the progression of systemic diseases, while systemic diseases can, in turn, worsen the course of periodontitis [9-11]. The pathogenesis of periodontitis is complex and multifactorial, primarily involving infection, inflammatory responses, abnormal immune regulation, and bone destruction [12, 13]. Pathogenic microorganisms in dental plaque, such as *Porphyromonas gingivalis* and *Fusobacterium nucleatum*, are key initiating factors in periodontitis [14-16]. These pathogens induce an excessive host immune response by producing endotoxins and other harmful metabolites, promoting chronic inflammation of the periodontal tissues. During the inflammatory process, large amounts of inflammatory factors, such as TNF-α, IL-1β, and IL-6, are released, further exacerbating local tissue destruction, particularly the resorption of alveolar bone [17-19]. Moreover, there is a close bidirectional relationship between periodontitis and type 2 diabetes [20]. Type 2 diabetes not only promotes the onset and progression of periodontitis but also makes patients more susceptible to severe periodontal tissue destruction [21-23]. Conversely, periodontitis can affect blood sugar control, and the systemic effects of inflammation can increase insulin resistance, thereby exacerbating blood sugar fluctuations [24, 25]. Additionally, uncontrolled periodontitis may increase the risk of chronic complications in patients with type 2 diabetes, including cardiovascular disease, kidney disease, and retinopathy [26]. Effectively controlling periodontitis is not only crucial for oral health but also has a profound impact on overall systemic health. Current treatment methods for periodontitis primarily include mechanical removal of dental plaque and pharmacotherapy [27, 28]. However, mechanical treatment is often limited in patients with deep periodontal pockets and complex root structures [29], while the use of local antibiotics is often accompanied by issues such as bacterial resistance and imbalance of the oral microbiota [30]. Therefore, although current treatment measures can temporarily control the progression of the disease, their long-term efficacy is limited, and new treatments are urgently needed. Traditional Chinese Medicine (TCM), as an alternative treatment method, has shown great potential in the treatment of periodontitis in recent years [31-33]. TCM, characterized by its multi-target and multi-pathway effects, offers a promising alternative to traditional treatments when integrated into periodontal care, potentially overcoming the limitations of traditional therapies and improving overall patient outcomes.

*Smilax glabra* Roxb is a well-known TCM, widely used around the world for its remarkable pharmacological activities. Its main chemical constituents include flavonoids, phenylpropanoids, and phenolic acids, with astilbin considered the primary bioactive component. Modern pharmacological studies have demonstrated that SGR extracts possess various biological activities, including anti-inflammatory, immunomodulatory, antibacterial, antioxidant, and hepatoprotective effects. Notably, SGR extract has shown significant anti-inflammatory effects in the treatment of chronic inflammation-related diseases, such as intestinal inflammation, arthritis, or chronic nephritis [34]. Studies have demonstrated that SGR extract can regulate inflammatory responses by inhibiting the release of pro-inflammatory cytokines, such as TNF-α, IL-1β, and IL-6 induced by LPS [35]. Additionally, its antioxidant activity can reduce the production of Reactive Oxygen Species (ROS), thereby minimizing tissue damage caused by inflammation. In terms of immunoregulation, SGR can modulate the immune system through multiple pathways, particularly by regulating overactivated macrophages and dysfunctional T lymphocytes, to reduce abnormal immune activity during inflammation [34]. Based on the pharmacological properties of SGR and its extracts, it is likely to be a promising therapeutic agent for the treatment of periodontitis. Although the efficacy of SGR in other inflammation-related diseases has been widely studied and reported, no studies currently exist regarding its application in the treatment of periodontitis. Therefore, this study will use network pharmacology methods to analyze the interactions between the active components of SGR and periodontitis-related targets and pathways, aiming to elucidate its potential therapeutic mechanisms.

## 2 METHODS

### 2.1 Smilax Glabra Roxb Active Components Screening and Target Prediction

The Traditional Chinese Medicine Systems Pharmacology Database and Analysis Platform (TCMSP, https://old.tcmsp-e.com/tcmsp.php) is a database and analysis platform specifically designed for studying traditional Chinese medicines. It integrates information on chemical components, targets, related diseases, and pharmacological activities of traditional Chinese medicines. To identify the active components of SGR, we used “Tufuling” as the search query in the TCMSP database. Components that met the pharmacokinetic criteria of Oral Bioavailability (OB) ≥ 30% and Drug-Likeness (DL) ≥ 0.18 were selected for further analysis. For these active compounds, we first retrieved their molecular formulas and SMILES structures from the PubChem database (https://pubchem.ncbi.nlm.nih.gov/). Following previously published methodologies [36, 37], we integrated three databases-TCMSP, STITCH (http://stitch.embl.de/), and SwissTargetPrediction (http://swisstargetprediction.ch/)-to predict potential targets. This multi-database integration strategy allows for data complementarity and redundancy verification, thereby effectively reducing reliance on any single database. During the search, the species was set to “*Homo sapiens*” with a confidence score ≥ 0.4 and a probability > 0, while all other settings were kept at their default values.

### 2.2 Screening of Periodontitis Disease Targets

The GeneCards (https://www.genecards.org/), Therapeutic Target Database (TTD, http://db.idrblab.net/ttd/), and Online Mendelian Inheritance in Man (OMIM, https://omim.org/) databases were used to search for gene targets associated with periodontitis. “Periodontitis” was used as the search term, with a relevance score >3, and the retrieved genes were defined as periodontitis-related target genes. The Venny 2.1.0 software was used to identify overlapping genes between the active ingredient targets of SGR and periodontitis-related targets, which were considered the potential key targets of SGR in treating periodontitis.

### 2.3 Construction of the Protein-Protein Interaction Network of Overlapping Genes

The STRING database (http://string-db.org) was used to construct the Protein-Protein Interaction network of overlapping genes. During the database search, the species was set to “*Homo sapiens*”, the confidence score was set to 0.7, and other settings were left at their default values. The PPI interaction network was visualized using Cytoscape software. Additionally, the MCODE algorithm was used to construct subnetworks, and topological analysis of the subnetworks was performed using the cytoNCA plugin, which included calculating Betweenness Centrality (BC), Closeness Centrality (CC), and Degree (De).

### 2.4 GO and KEGG Pathway Enrichment Analysis of Overlapping Genes

To explore the potential biological functions and pathways of the intersection genes between the active components of SGR and periodontitis-related target genes, we used the Database for Annotation, Visualization, and Integrated Discovery (DAVID, https://david.ncifcrf.gov/home.jsp) to conduct GO and KEGG pathway enrichment analyses on these overlapping genes.

### 2.5 Construction of Component-Target-Pathway Interaction Network

To reveal how the active components of SGR regulate disease-related signaling pathways through targets, the top 20 pathways from the KEGG enrichment analysis were selected and visualized in Cytoscape software, along with the intersection targets and active components. In the network, nodes represent genes and compounds, while edges represent the interactions between genes and compounds. The number of edges connected to a target gene or compound indicates its degree in the network; the larger the degree, the larger the node, highlighting the importance of the compound and pathway in the treatment of periodontitis by SGR.

### 2.6 Molecular Docking

To further validate the interaction between the active components of SGR and the potential key targets for periodontitis, molecular docking analysis was performed. First, the key targets identified from the overlapping genes and their corresponding active components were selected. The structures of the target proteins and active components were downloaded from the RCSB PDB database and the PubChem database, respectively. Using PyMOL software, the proteins and ligands were prepared by removing water molecules and ligands from the proteins, and hydrogen atoms were added. The ligand-target protein docking simulation was run using AutoDock Vina software, and the pose with the lowest binding free energy was selected as the optimal binding mode. The interactions between the ligands and target proteins were further analyzed using PyMOL.

## 3 RESULTS

### 3.1 Acquisition of Active Components and Overlapping Targets

Using the TCMSP database, 15 active compounds from SGR were selected based on the criteria of OB ≥ 30% and DL ≥ 0.18. These compounds were mainly terpenes and flavonoids (Table **1**). In the TCMSP, STITCH, and SwissTargetPrediction databases, a total of 527 targets were predicted for the 15 active components. Subsequently, 367 periodontitis-related targets were identified using the GeneCards, TTD, and OMIM databases. Through Venny software, 75 overlapping targets (Fig. **S1**) were identified between periodontitis and the active components of SGR, which are considered potential targets for the treatment of periodontitis with SGR.

### 3.2 PPI Analysis and Identification of Hub Genes

**Fig. (1):**
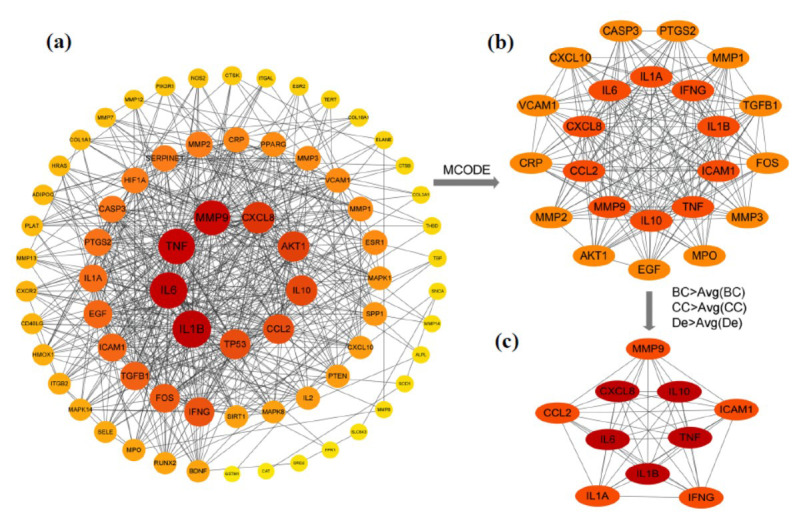
Modular analysis of PPI network. (**a**) The PPI network of potential targets of Smilax glabra Roxb in periodontitis. (**b**) PPI subnetwork obtained *via* module analysis using the MCODE algorithm. (**c**) Select the targets in the sub-network where BC, CC, and De are all greater than the average values, and further represent the targets in red where the Degree is greater than the top 50% of targets. The darker the color, the more significant the gene. **Abbreviation**: BC, betweenness centrality; CC, closeness centrality; De, degree. The size of the dots depends on the degree value.

To construct the potential protein-protein interaction network, 75 overlapping targets were imported into the String database and Cytoscape 3.8 software. The topological analysis of the PPI network revealed 71 nodes and 551 edges (Fig. **1a**). In the network, the larger and darker a node, the greater its degree value, indicating its importance in the PPI network. Hub genes were screened using network topology parameters (Table **2**). In the first step, the MCODE algorithm in Cytoscape was used to identify module networks within the PPI network, with the default parameters set as follows: Degree Cutoff = 2, Node Score Cutoff = 0.2, k-Core = 2, and Max. Depth = 100. The resulting module network contained 23 nodes and 194 edges, with a score of 17.636 (Fig. **1b**). This helps us to understand the functional core of the PPI network, which is of great significance for deciphering complex biological processes and identifying potential therapeutic targets. Within the module network, targets with higher degree values included IL6, IL1B, TNF, IL10, CXCL8, IFNG, MMP9, IL1A, CCL2, and ICAM1. In the second step, hub genes were selected based on BC, CC, and De values greater than the average (Fig. **1c**). Finally, genes with a degree value higher than the top 50% were selected [38]. As a result, 5 hub genes were identified: IL-6, IL-1β, TNF, IL-10, and CXCL8.

### 3.3 GO Functional Enrichment Analysis

**Fig. (2):**
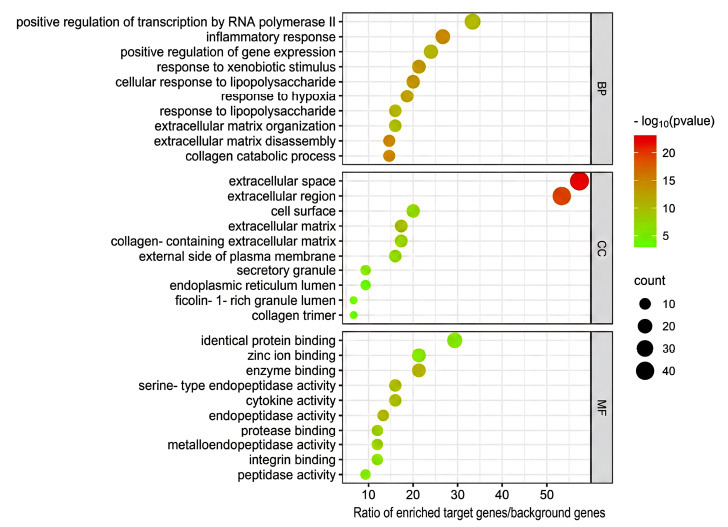
GO analysis of the intersection targets. The X-axis represents the ratio of enriched target genes/background genes. Node color is displayed in a gradient from red to green in descending order of the *P*-value. The size of the nodes is arranged in ascending order of the number of genes.

Based on the 75 intersecting target genes, a total of 569 GO terms were obtained, including 460 biological processes, 46 cellular components, and 63 molecular functions. The top 10 terms in each category with *p*-values less than 0.05 were visualized (Fig. **2**). The results indicated that SGR mainly acts on processes such as the extracellular space and extracellular region, influencing biological processes like the positive regulation of transcription through RNA polymerase II, inflammatory response, and the positive regulation of gene expression, thereby affecting identical protein binding, enzyme binding, and zinc ion binding.

### 3.4 KEGG Pathway Enrichment Analysis

**Fig. (3):**
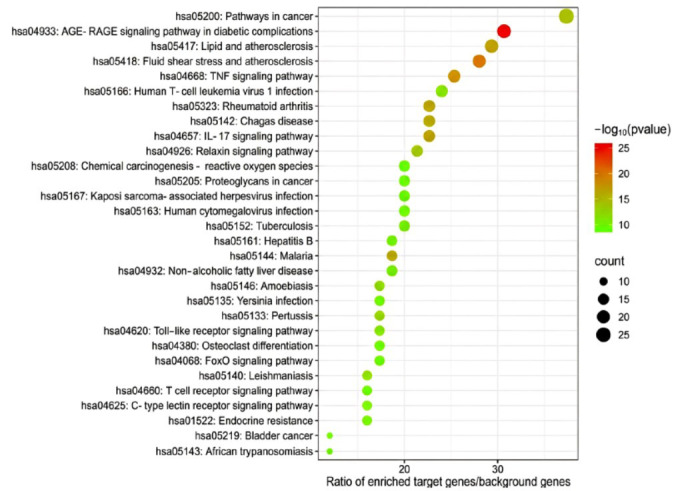
KEGG enrichment analysis of the intersection targets. The X-axis represents the ratio of enriched target genes/background genes. Node color is displayed in a gradient from red to green in descending order of the *P*-value. The size of the nodes is arranged in ascending order of the number of genes.

The KEGG enrichment analysis identified a total of 153 signaling pathways, among which 145 pathways had *p*-values less than 0.05. We further applied False Discovery Rate (FDR) correction, and 145 pathways remained significantly enriched (FDR < 0.05) (Table **S1**). The top 30 pathways were visualized in a bubble chart (Fig. **3**). The results indicated that the potential targets of SGR were primarily enriched in pathways such as the AGE-RAGE signaling pathway, the lipid and atherosclerosis pathway, and the TNF signaling pathway, suggesting that SGR may exert its therapeutic effects on periodontitis by modulating diabetes-related and inflammatory pathways.

### 3.5 Construction of the *Smilax Glabra* Roxb Active Component-Target-Pathway Network

**Fig. (4):**
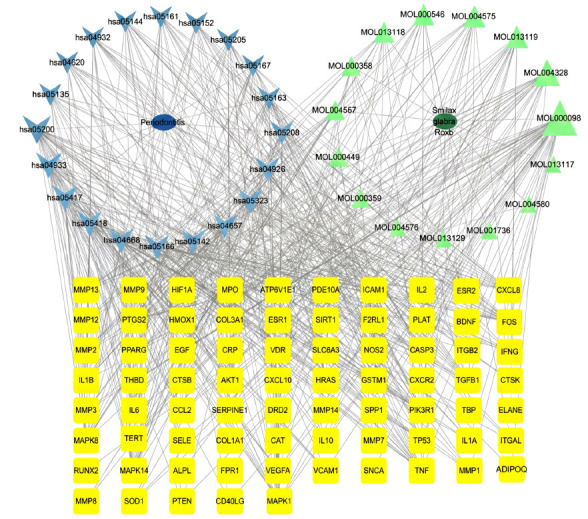
Component-target-pathway network. The green triangles represent the compounds; the yellow squares represent the genes; the blue arrows represent the pathways; and the edges represent the interactions. The line between two nodes represents the interaction, and the size of each node indicates the number of connections.

The top 20 KEGG pathways identified through the enrichment analysis were selected as periodontitis-related pathways. Along with the active components and overlapping targets, these pathways were imported into Cytoscape software to construct the “drug-component-target-pathway” network (Fig. **4**). In the network, yellow square nodes represent the overlapping gene targets, blue V-shaped nodes represent pathways related to potential targets, and green triangular nodes represent the active components of SGR. The Network Analyzer plugin analysis revealed that the network consisted of 112 nodes and 528 edges. The larger a node, the greater its degree value, indicating a significant regulatory role in the entire network. Compounds with high degree values, such as quercetin, naringenin, enhydrin, astilbin, and diosgenin, were closely connected to 20 pathways, suggesting they may be the primary active components of SGR in the treatment of periodontitis.

### 3.6 Molecular Docking

Based on the hub genes identified in the PPI network, key targets were further investigated. TNF (PDB: 7JRA), IL1B (PDB: 5R7W), and IL6 (PDB: 1ALU) were selected as docking targets, and their corresponding active components were used as ligands. The overactivation of the AGE-RAGE pathway is considered a central pathological mechanism in diabetes-associated periodontitis [39, 40], playing a key role in the production and regulation of inflammatory factors, such as TNF-α, IL-1β, and IL-6. Based on the KEGG results, RAGE (PDB: 2MOV) was also selected for molecular docking with key active components quercetin and astilbin (Table **3**).

**Fig. (5):**
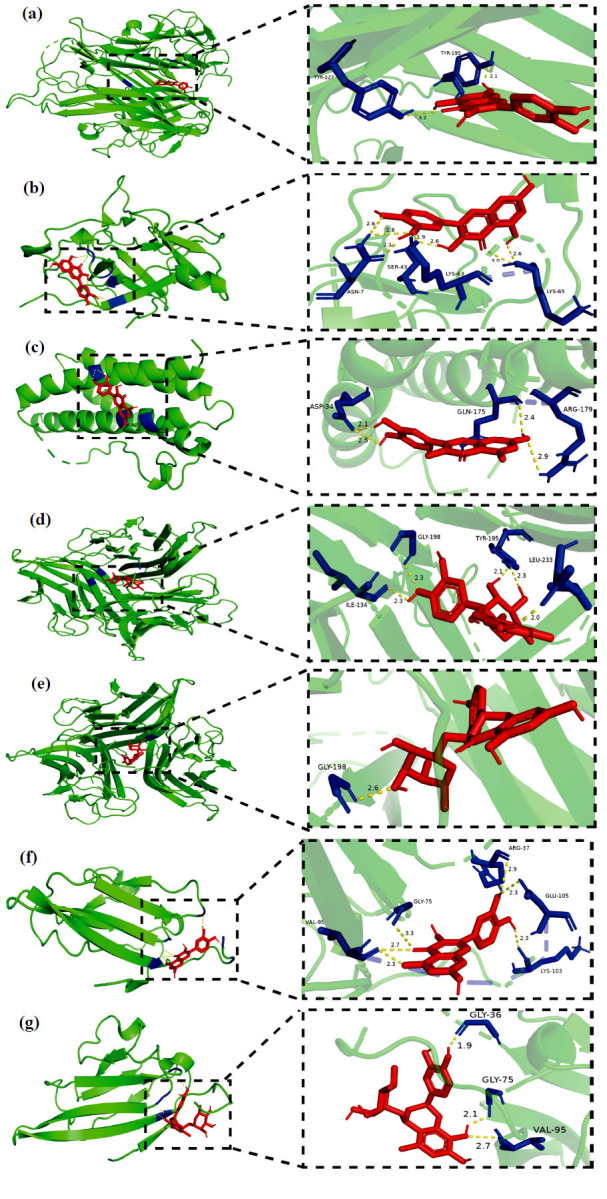
Molecular docking of key targets and core components. (**a**) Molecular docking of TNF and quercetin. (**b**) Molecular docking of IL1B and quercetin. (**c**) Molecular docking of IL6 and quercetin. (**d**) Molecular docking of TNF and astilbin. (**e**) Molecular docking of TNF and Neoastilbin. (**f**) Molecular docking of RAGE and quercetin. (**g**) Molecular docking of RAGE and astilbin.

The results showed that core targets had strong binding activity with their respective active components. Specifically, molecular docking analysis showed that quercetin formed hydrogen bonds with Tyr227 and Tyr195 in TNF (Fig. **5a**). In addition, quercetin interacted with IL1B by forming hydrogen bonds with Asn7, Ser43, and Lys63 and 65 (Fig. **5b**). When docked with IL6, quercetin established stable hydrogen bonds with Asp34, Gln175, and Arg179 (Fig. **5c**). The docking model of astilbin with TNF revealed the formation of multiple hydrogen bonds with Ile134, Gly198, Tyr195, and Leu233 (Fig. **5d**). Neoastilbin mainly interacted with Gly198 in TNF through hydrogen bonding (Fig. **5e**). Furthermore, quercetin and astilbin also exhibited strong binding activity with target proteins in the pathway. The docking model of quercetin with RAGE indicated hydrogen bond formation with Val95, Gly75, Arg37, Glu105, and Lys103 (Fig. **5f**). Astilbin primarily formed hydrogen bonds with Gly36, Gly75, and Val95 in RAGE (Fig. **5g**), demonstrating the multi-pathway, multi-target, and multi-component characteristics of traditional Chinese medicine in treating diseases.

## 4 DISCUSSION

Periodontitis is a chronic inflammatory disease characterized by the destruction of periodontal supporting tissues, including the gums, periodontal ligaments, and alveolar bone. The impact of periodontitis is not limited to oral health, as it is closely linked to various systemic diseases, such as diabetes and cardiovascular diseases [41-43]. Surveys show that the global prevalence of advanced periodontitis in adults is approximately 5-15% [44-46], with the prevalence of severe periodontitis among Asians ranging from 15-20% [47], which poses a severe burden on global public health. However, current treatment methods for periodontitis, such as mechanical debridement, anti-inflammatory drugs, antibiotics, and surgical treatment, have not significantly improved outcomes and are often accompanied by certain side effects.

For a long time, herbal medicine has been widely used to treat periodontal diseases, and its effectiveness has been confirmed in many studies. There is ample evidence that natural active ingredients, such as plant extracts, can effectively alleviate the symptoms of periodontitis and improve patients' oral health [48-51]. *Smilax glabra* Roxb, as a traditional Chinese medicine, has attracted significant attention due to its remarkable anti-inflammatory, antioxidant, and immune-regulating properties. In a study using a psoriasis mouse model, SGR was found to reduce the expression of inflammatory factors, such as IL-6, IL-17, TNF-α, and IL-1β, and significantly downregulate the proportion of Th1 and Th17 cells, thereby correcting the expression of immune cell subsets and regulating abnormal immune responses [52]. Other studies have shown that phenolic-enriched extracts from the rhizomes of SGR as well as two polysaccharide components, can significantly inhibit the production of NO, IL-6, and TNF-α in LPS-induced RAW264.7 cells [53, 54]. Th1 and Th17 cells exacerbate the destruction of periodontal tissues by releasing pro-inflammatory cytokines, such as IL-1, TNF-α, and IL-6, which induce bone resorption and destroy alveolar bone. Therefore, the immune-regulating and anti-inflammatory activities of SGR provide scientific support for its potential use in the treatment of periodontitis. In an ovariectomized mouse model of osteoporosis, astilbin, an extract from SGR, was shown to inhibit osteoclast formation and reduce bone resorption, indicating that SGR may have beneficial effects on osteolytic diseases [55]. Moreover, the imbalance between antioxidant protection and the production of ROS has been shown to play a role in the pathogenesis of periodontitis. SGR has strong antioxidant activity, primarily through the scavenging of free radicals and chelation of metal ions [56]. Due to its multiple pharmacological mechanisms, SGR has been widely used in the treatment of other systemic inflammatory or chronic diseases. Periodontitis is considered a major complication of diabetes, and its severity is positively correlated with poor blood sugar control. Studies have shown that in diabetic mice, SGR can improve insulin sensitivity and lower blood glucose levels, thereby helping to counteract diabetes and obesity [57, 58]. Overall, SGR exerts multiple effects on the key pathogenic mechanisms of periodontitis, making it a promising potential treatment for the disease.

To further explore this hypothesis, we used network pharmacology to investigate the mechanisms of SGR in the treatment of periodontitis. In our study, through the analysis of the PPI network of overlapping targets between SGR and periodontitis, we identified a module network consisting of 23 targets and five core target genes. The genes within the module network are mainly involved in regulating the TNF signaling pathway and the IL-17 signaling pathway, thereby promoting the progression of periodontitis by inducing the release of pro-inflammatory cytokines, recruiting immune cells, and disrupting bone metabolism. The core target genes encode the proteins IL-6, IL-1β, TNF-α, IL-10, and IL-8. Among them, IL-6, IL-1β, and TNF-α are the main pro-inflammatory factors in the local tissues of periodontitis [59], while IL-8—a key chemokine—attracts inflammatory cells to the site of inflammation. IL-10 primarily participates in anti-inflammatory processes, reflecting the potential of SGR in regulating the inflammatory response through the interaction of multiple inflammatory mediators to achieve therapeutic effects against periodontitis. Further KEGG enrichment analysis of the overlapping targets also identified several inflammatory pathways, including the TNF signaling pathway, IL-17 signaling pathway, and Rheumatoid arthritis pathway. Additionally, highly enriched pathways included the AGE-RAGE signaling pathway in diabetic complications and the lipid and atherosclerosis pathway. This suggests that, in addition to inhibiting pro-inflammatory factors and regulating immune responses to alleviate local inflammation, SGR may also provide more comprehensive therapeutic effects by affecting systemic inflammatory and metabolic pathways. Advanced Glycation End-products (AGEs) are glycosylated products generated in a high-glucose environment and are widely present in patients with diabetes [60-62]. AGEs interact with the Receptor for Advanced Glycation End-products (RAGE) to activate downstream signaling pathways, leading to an increase in oxidative stress and inflammatory responses [63-65]. This process plays an important role in diabetes and its complications. As a common complication of diabetes, the severity of periodontitis is closely related to the patient's blood sugar control levels. Excessive production of AGEs not only occurs in diabetes-related organs but also accumulates in periodontal tissues. Studies have shown that RAGE expression is significantly elevated in the periodontal tissues of periodontitis patients with type 2 diabetes [65-67]. The AGE-RAGE signaling pathway can activate downstream pathways, such as NF-κB and MAPK, leading to the upregulation of inflammatory mediators, such as IL-1β, TNF-α, and IL-6 in periodontal tissues, and enhancing oxidative stress in certain cells [68-71]. AGEs can also increase the expression of Matrix Metalloproteinase 1 (MMP-1) in Human Gingival Fibroblasts (HGFs), while decreasing alkaline phosphatase activity and osteocalcin expression in osteoblasts [72, 73]. This suggests that AGEs can exacerbate the inflammatory response in diabetic periodontitis, promote the destruction of gingival and alveolar bone, and accelerate the progression of periodontitis. Molecular docking allows the prediction of protein-ligand binding interactions at the atomic level. Generally, a binding energy < 0 indicates that the ligand can spontaneously bind to the receptor protein; a binding energy < -5.0 kcal/mol suggests good binding affinity; and a binding energy < -7.0 kcal/mol is typically considered indicative of strong binding ability [74]. Additionally, other studies have proposed a threshold of ≤ -5.0 kcal/mol as indicative of potential bioactivity [75]. In this study, all core active compounds of Smilax glabra exhibited binding energies below -5.0 kcal/mol with the key therapeutic targets for periodontitis, suggesting favorable binding affinity. The lowest binding energy observed was -6.9 kcal/mol, indicating that these compounds may possess definite target-specific potential. Moreover, several compounds exhibited strong affinities with key inflammatory targets, such as TNF. Previous studies have reported that known TNF-α inhibitors (*e.g*., SPD304, UCB-6876, and UCB-9260) exhibit binding energies ranging from -7.1 to -9.2 kcal/mol. In our study, quercetin, astilbin, and neoastilbin exhibited binding energies of -8.5, -7.4, and -7.3 kcal/mol with TNF, respectively, which are comparable to or even better than those of known inhibitors, indicating their potential anti-inflammatory activity. These findings suggest that the multi-component nature of *Smilax glabra* may contribute to synergistic effects in enhancing anti-inflammatory efficacy. In addition, the key active components of SGR exhibited strong binding activity with RAGE, indicating that SGR may effectively act on the AGE-RAGE pathway. Therefore, SGR has particular advantages in treating patients with systemic diseases, such as diabetes and cardiovascular diseases. Compared to antibiotics, SGR can avoid issues such as microbial dysbiosis and antibiotic resistance, addressing the limitations of treatment options for these patients.

## CONCLUSION

In summary, this study utilized network pharmacology and molecular docking approaches to elucidate the multi-target synergistic mechanisms of SGR in the treatment of periodontitis. A total of 15 active compounds were identified from SGR, and 75 core targets associated with periodontitis were screened. Key targets, such as IL-6, IL-1β, TNF, IL-10, and CXCL8, are primarily involved in regulating inflammatory cytokines and immune responses. GO enrichment analysis revealed that SGR mainly affects the function of the extracellular space, RNA polymerase II-mediated transcription, and inflammatory processes. In terms of molecular function, zinc ion binding and regulation of enzymatic activity were prominent. KEGG pathway analysis revealed that SGR may exert therapeutic effects through modulation of the AGE-RAGE, TNF, and IL-17 signaling pathways, thereby not only attenuating local inflammation in periodontal tissues but also influencing diabetes-related pathological processes. Molecular docking analysis confirmed that the core compounds exhibit strong binding affinities to their respective targets. Quercetin was shown to form stable hydrogen bonds with TNF, IL1B, and RAGE, with binding energies superior to those of some known inhibitors. Astilbin also demonstrated high binding affinity with TNF and RAGE, highlighting the synergistic interactions among multiple components of SGR. These findings suggest that SGR holds therapeutic promise for the treatment of periodontitis. For instance, Smilax glabra can suppress pro-inflammatory cytokines, such as IL-6 and TNF-α, and help restore immune homeostasis. Moreover, it modulates the AGE-RAGE signaling pathway to alleviate oxidative stress and bone destruction in diabetic periodontitis. The multi-target actions of natural compounds may help reduce the risk of antibiotic resistance and are particularly beneficial for patients with systemic comorbidities.

Despite these promising results, several limitations remain. It should be noted that the STITCH database (version 5.0) used in this study is no longer maintained by its original development team, which may result in the exclusion of some recently discovered compound-target interactions. To reduce potential bias, a multi-database cross-validation strategy was employed in this study. Nonetheless, further studies incorporating data from single-cell transcriptomics, epigenomics, and the oral microbiome are warranted to gain a more comprehensive understanding of the regulatory effects of SGR on periodontitis. In addition, the active ingredients and key targets identified through network pharmacology have yet to be validated *in vitro* or *in vivo*. The binding affinities predicted by molecular docking should be further confirmed by experimental methods, such as protein-protein interaction assays. These experimental validations will be the focus of our future work, providing a theoretical and empirical foundation for the clinical application of SGR in periodontitis therapy.

## AUTHORS’ CONTRIBUTIONS

The authors confirm their contribution to the paper as follows: Study concept and design: XW, Methodology: NL, Data analysis or interpretation: PM, Visualization: YZ, PZ, CT, YC, Draft manuscript: JH, All authors read and approved the submitted version.

## Figures and Tables

**Table 1:** Active compounds in Smilax glabra Roxb.

**Mol ID**	**PubChem ID**	**Molecule Name**	**MW**	**OB(%)**	**DL**	**Type**
MOL004575	119258	Astilbin	450.43	36.46	0.74	Flavonoids
MOL013118	442437	Neoastilbin	450.43	40.54	0.74	Flavonoids
MOL004567	101937309	Isoengelitin	434.43	34.65	0.7	Flavonoids
MOL004580	443758	cis-Dihydroquercetin	304.27	66.44	0.27	Flavonoids
MOL001736	712316	(-)-taxifolin	304.27	60.51	0.27	Flavonoids
MOL013129	5320468	(2R,3R)-2-(3,5-dihydroxyphenyl)-3,5,7-trihydroxychroman-4-one	304.27	63.17	0.27	Flavonoids
MOL013119	5281441	Enhydrin	464.51	40.56	0.74	Terpenoid
MOL004576	439533	Taxifolin	304.27	57.84	0.27	Flavonoids
MOL000098	5280343	Quercetin	302.25	46.43	0.28	Flavonoids
MOL004328	439246	Naringenin	272.27	59.29	0.21	Flavonoids
MOL013117	129394	4,7-Dihydroxy-5-methoxyl-6-methyl-8-formyl-flavan	314.36	37.03	0.28	Flavonoids
MOL000546	99474	Diosgenin	414.69	80.88	0.81	Terpenoid
MOL000359	12303645	Sitosterol	414.79	36.91	0.75	Terpenoid
MOL000358	222284	Beta-sitosterol	414.79	36.91	0.75	Terpenoid
MOL000449	5280794	Stigmasterol	412.77	43.83	0.76	Terpenoid

**Table 2:** Topological parameters of the PPI subnetwork.

**Gene**	**Name**	**Betweenness**	**Closeness**	**Degree**
IL6	Interleukin-6	12.13320568	1	22
IL1B	Interleukin-1 beta	12.13320568	1	22
TNF	Tumor necrosis factor	12.13320568	1	22
IL10	Interleukin-10	12.13320568	1	22
CXCL8	Interleukin-8	12.13320568	1	22
IFNG	Interferon gamma	10.21031191	0.956521739	21
MMP9	Matrix metalloproteinase-9	10.18556444	0.956521739	21
IL1A	Interleukin-1 alpha	8.796941947	0.916666667	20
CCL2	C-C motif chemokine 2	6.226112776	0.88	19
ICAM1	Intercellular adhesion molecule 1	5.155455655	0.846153846	18

**Table 3:** The molecular docking binding energies between active components and core targets.

**Molecular Name**	**Target**	**PDB ID**	**Affinity (kcal/mol)**
Quercetin	TNF	7JRA	-8.5
Quercetin	IL1B	5R7W	-6.9
Quercetin	IL6	1AlU	-7
Quercetin	RAGE	2MOV	-6.9
Astilbin	TNF	7JRA	-7.4
Astilbin	RAGE	2MOV	-7.1
Neoastilbin	TNF	7JRA	-7.3

## Data Availability

All data generated or analyzed during this study are included in this published article.

## LIST OF ABBREVIATIONS

AGEs: Advanced Glycation End-products
BC: Betweenness Centrality
CC: Closeness Centrality
CCL2: C-C Motif Chemokine 2
CXCL8: Interleukin-8
De: Degree
DL: Drug-likeness
HGFs: Human Gingival Fibroblasts
ICAM1: Intercellular Adhesion Molecule 1
IFNG: Interferon Gamma
IL10: Interleukin-10
IL1α: Interleukin-1 Alpha
IL1β: Interleukin-1 Beta
IL6: Interleukin-6
KEGG: Kyoto Encyclopedia of Genes and Genomes
MAPK: Mitogen-activated Protein Kinase
MMP-1: Matrix Metalloproteinase 1
MMP9: Matrix Metalloproteinase-9
NF-κB: Nuclear Factor Kappa-B
OB: Oral Bioavailability
RAGE: Receptor for Advanced Glycation End-products
ROS: Reactive Oxygen Species
SGR: *Smilax Glabra* Roxb
TCM: Traditional Chinese Medicine
Th1: T Helper Type 1
Th17: T Helper Type 17
TNF: Tumor Necrosis Factor
