# Supplementary material for: Exploring the Potential Mechanism of Smilax Glabra Roxb in Periodontitis Through Network Pharmacology and Molecular Docking
Source: Curr Pharm Des. 2025 Aug 8;32(17):1362–73. doi: 10.2174/0113816128391490250729102829 (PMC13312393; doi:10.2174/0113816128391490250729102829)
Supplement: Supplementary file 1 [file CPD-32-17-1362_SD1.pdf]

Supplementary Material

Exploring the Potential Mechanism of *Smilax Glabra* Roxb in Periodontitis Through Network Pharmacology and Molecular Docking

Jinjia Hong[1,2,#], Peilun Ma[1,2,#], Yuan Zhang[1,2], Na Li[1,2], Pengfei Zhang[1,2], Chunrui Tian[1,2], Yukang Cao[1,2] and Xing Wang[1,2,*]

[1]Shanxi Medical University School and Hospital of Stomatology, Taiyuan, 030001, China; [2]Shanxi Province Key Laboratory of Oral Diseases Prevention and New Materials, Taiyuan, 030001, China

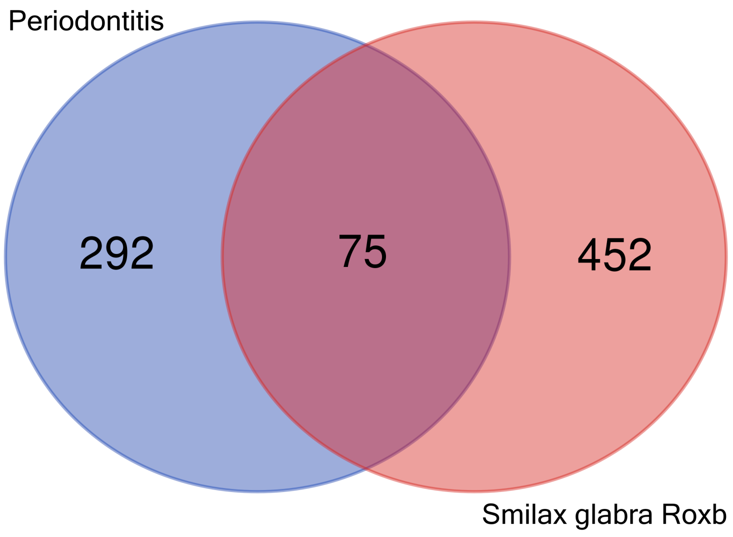

Fig. (1). Venn diagram for disease targets of periodontitis and active targets of Smilax glabra Roxb.

1873-4286/26 © 2026 Bentham Science Publishers

Table S1. FDR correction of KEGG enrichment analysis results for intersection targets.

Category	Term	Count	%	PValue	FDR
KEGG_PATHWAY	hsa04933:AGE-RAGE signaling pathway in diabetic complications	23	30.66666667	1.30E-26	1.09E-24
KEGG_PATHWAY	hsa05418:Fluid shear stress and atherosclerosis	21	28	3.14E-20	1.32E-18
KEGG_PATHWAY	hsa04668:TNF signaling pathway	19	25.33333333	9.33E-19	2.61E-17
KEGG_PATHWAY	hsa05417:Lipid and atherosclerosis	22	29.33333333	9.41E-18	1.98E-16
KEGG_PATHWAY	hsa05323:Rheumatoid arthritis	17	22.66666667	1.37E-17	2.30E-16
KEGG_PATHWAY	hsa04657:IL-17 signaling pathway	17	22.66666667	1.64E-17	2.30E-16
KEGG_PATHWAY	hsa05144:Malaria	14	18.66666667	5.04E-17	6.05E-16
KEGG_PATHWAY	hsa05142:Chagas disease	17	22.66666667	6.35E-17	6.66E-16
KEGG_PATHWAY	hsa05200:Pathways in cancer	28	37.33333333	1.46E-15	1.36E-14
KEGG_PATHWAY	hsa04926:Relaxin signaling pathway	16	21.33333333	6.53E-14	5.48E-13
KEGG_PATHWAY	hsa05133:Pertussis	13	17.33333333	7.83E-13	5.98E-12
KEGG_PATHWAY	hsa05166:Human T-cell leukemia virus 1 infection	18	24	1.14E-12	8.01E-12
KEGG_PATHWAY	hsa05140:Leishmaniasis	12	16	1.76E-11	1.14E-10
KEGG_PATHWAY	hsa05146:Amoebiasis	13	17.33333333	2.36E-11	1.41E-10
KEGG_PATHWAY	hsa04620:Toll-like receptor signaling pathway	13	17.33333333	4.66E-11	2.61E-10
KEGG_PATHWAY	hsa05152:Tuberculosis	15	20	1.11E-10	5.83E-10
KEGG_PATHWAY	hsa04932:Non-alcoholic fatty liver disease	14	18.66666667	2.54E-10	1.25E-09
KEGG_PATHWAY	hsa01522:Endocrine resistance	12	16	2.90E-10	1.35E-09
KEGG_PATHWAY	hsa05167:Kaposi sarcoma-associated herpesvirus infection	15	20	3.46E-10	1.53E-09
KEGG_PATHWAY	hsa05143:African trypanosomiasis	9	12	3.70E-10	1.56E-09
KEGG_PATHWAY	hsa05161:Hepatitis B	14	18.66666667	4.06E-10	1.62E-09
KEGG_PATHWAY	hsa04068:FoxO signaling pathway	13	17.33333333	4.49E-10	1.72E-09
KEGG_PATHWAY	hsa04625:C-type lectin receptor signaling pathway	12	16	5.52E-10	2.02E-09
KEGG_PATHWAY	hsa05205:Proteoglycans in cancer	15	20	5.88E-10	2.06E-09
KEGG_PATHWAY	hsa05135:Yersinia infection	13	17.33333333	7.55E-10	2.54E-09
KEGG_PATHWAY	hsa05219:Bladder cancer	9	12	8.94E-10	2.89E-09
KEGG_PATHWAY	hsa04380:Osteoclast differentiation	13	17.33333333	1.05E-09	3.27E-09
KEGG_PATHWAY	hsa05208:Chemical carcinogenesis - reactive oxygen species	15	20	2.26E-09	6.54E-09
KEGG_PATHWAY	hsa05163:Human cytomegalovirus infection	15	20	2.26E-09	6.54E-09
KEGG_PATHWAY	hsa04660:T cell receptor signaling pathway	12	16	2.79E-09	7.82E-09
KEGG_PATHWAY	hsa04218:Cellular senescence	13	17.33333333	3.35E-09	9.08E-09
KEGG_PATHWAY	hsa05164:Influenza A	13	17.33333333	9.48E-09	2.49E-08
KEGG_PATHWAY	hsa04066:HIF-1 signaling pathway	11	14.66666667	1.32E-08	3.36E-08
KEGG_PATHWAY	hsa05145:Toxoplasmosis	11	14.66666667	1.57E-08	3.89E-08

Category	Term	Count	%	PValue	FDR
KEGG_PATHWAY	hsa05210:Colorectal cancer	10	13.33333333	2.57E-08	6.18E-08
KEGG_PATHWAY	hsa05235:PD-L1 expression and PD-1 checkpoint pathway in cancer	10	13.33333333	3.48E-08	8.11E-08
KEGG_PATHWAY	hsa05171:Coronavirus disease - COVID-19	14	18.66666667	4.09E-08	9.29E-08
KEGG_PATHWAY	hsa05165:Human papillomavirus infection	16	21.33333333	4.29E-08	9.49E-08
KEGG_PATHWAY	hsa05215:Prostate cancer	10	13.33333333	7.36E-08	1.59E-07
KEGG_PATHWAY	hsa04010:MAPK signaling pathway	15	20	8.36E-08	1.71E-07
KEGG_PATHWAY	hsa04917:Prolactin signaling pathway	9	12	8.22E-08	1.71E-07
KEGG_PATHWAY	hsa04210:Apoptosis	11	14.66666667	1.11E-07	2.22E-07
KEGG_PATHWAY	hsa04659:Th17 cell differentiation	10	13.33333333	1.72E-07	3.35E-07
KEGG_PATHWAY	hsa05321:Inflammatory bowel disease	8	10.66666667	7.67E-07	1.46E-06
KEGG_PATHWAY	hsa05162:Measles	10	13.33333333	1.48E-06	2.76E-06
KEGG_PATHWAY	hsa04936:Alcoholic liver disease	10	13.33333333	1.98E-06	3.62E-06
KEGG_PATHWAY	hsa05224:Breast cancer	10	13.33333333	2.49E-06	4.36E-06
KEGG_PATHWAY	hsa05212:Pancreatic cancer	8	10.66666667	2.45E-06	4.36E-06
KEGG_PATHWAY	hsa01521:EGFR tyrosine kinase inhibitor resistance	8	10.66666667	3.18E-06	5.45E-06
KEGG_PATHWAY	hsa04060:Cytokine-cytokine receptor interaction	13	17.33333333	3.72E-06	6.25E-06
KEGG_PATHWAY	hsa05169:Epstein-Barr virus infection	11	14.66666667	4.45E-06	7.28E-06
KEGG_PATHWAY	hsa05160:Hepatitis C	10	13.33333333	4.51E-06	7.28E-06
KEGG_PATHWAY	hsa05022:Pathways of neurodegeneration - multiple diseases	16	21.33333333	4.98E-06	7.90E-06
KEGG_PATHWAY	hsa04071:Sphingolipid signaling pathway	9	12	5.38E-06	8.36E-06
KEGG_PATHWAY	hsa04015:Rap1 signaling pathway	11	14.66666667	6.55E-06	1.00E-05
KEGG_PATHWAY	hsa04211:Longevity regulating pathway	8	10.66666667	7.02E-06	1.03E-05
KEGG_PATHWAY	hsa05213:Endometrial cancer	7	9.333333333	6.98E-06	1.03E-05
KEGG_PATHWAY	hsa05207:Chemical carcinogenesis - receptor activation	11	14.66666667	7.42E-06	1.08E-05
KEGG_PATHWAY	hsa05225:Hepatocellular carcinoma	10	13.33333333	7.79E-06	1.09E-05
KEGG_PATHWAY	hsa04370:VEGF signaling pathway	7	9.333333333	7.70E-06	1.09E-05
KEGG_PATHWAY	hsa04650:Natural killer cell mediated cytotoxicity	9	12	8.62E-06	1.19E-05
KEGG_PATHWAY	hsa05231:Choline metabolism in cancer	8	10.66666667	1.32E-05	1.79E-05
KEGG_PATHWAY	hsa04915:Estrogen signaling pathway	9	12	1.41E-05	1.85E-05
KEGG_PATHWAY	hsa04061:Viral protein interaction with cytokine and cytokine receptor	8	10.66666667	1.41E-05	1.85E-05
KEGG_PATHWAY	hsa04664:Fc epsilon RI signaling pathway	7	9.333333333	1.74E-05	2.25E-05
KEGG_PATHWAY	hsa05211:Renal cell carcinoma	7	9.333333333	1.89E-05	2.41E-05
KEGG_PATHWAY	hsa05230:Central carbon metabolism in cancer	7	9.333333333	2.06E-05	2.58E-05

Category	Term	Count	%	PValue	FDR
KEGG_PATHWAY	hsa05202:Transcriptional misregulation in cancer	10	13.33333333	2.16E-05	2.67E-05
KEGG_PATHWAY	hsa05218:Melanoma	7	9.333333333	2.41E-05	2.94E-05
KEGG_PATHWAY	hsa05332:Graft-versus-host disease	6	8	2.47E-05	2.96E-05
KEGG_PATHWAY	hsa04151:PI3K-Akt signaling pathway	13	17.33333333	2.67E-05	3.16E-05
KEGG_PATHWAY	hsa05132:Salmonella infection	11	14.66666667	2.86E-05	3.34E-05
KEGG_PATHWAY	hsa05214:Glioma	7	9.333333333	3.04E-05	3.50E-05
KEGG_PATHWAY	hsa04510:Focal adhesion	10	13.33333333	3.23E-05	3.65E-05
KEGG_PATHWAY	hsa04148:Efferocytosis	9	12	3.26E-05	3.65E-05
KEGG_PATHWAY	hsa05415:Diabetic cardiomyopathy	10	13.33333333	3.49E-05	3.86E-05
KEGG_PATHWAY	hsa04670:Leukocyte transendothelial migration	8	10.66666667	3.71E-05	4.05E-05
KEGG_PATHWAY	hsa04722:Neurotrophin signaling pathway	8	10.66666667	4.62E-05	4.97E-05
KEGG_PATHWAY	hsa05010:Alzheimer disease	13	17.33333333	5.68E-05	6.04E-05
KEGG_PATHWAY	hsa05134:Legionellosis	6	8	8.05E-05	8.46E-05
KEGG_PATHWAY	hsa05222:Small cell lung cancer	7	9.333333333	9.52E-05	9.88E-05
KEGG_PATHWAY	hsa04621:NOD-like receptor signaling pathway	9	12	1.27E-04	1.30E-04
KEGG_PATHWAY	hsa04213:Longevity regulating pathway - multiple species	6	8	1.31E-04	1.33E-04
KEGG_PATHWAY	hsa04613:Neutrophil extracellular trap formation	9	12	1.41E-04	1.41E-04
KEGG_PATHWAY	hsa04929:GnRH secretion	6	8	1.65E-04	1.65E-04
KEGG_PATHWAY	hsa05206:MicroRNAs in cancer	11	14.66666667	1.77E-04	1.77E-04
KEGG_PATHWAY	hsa04064:NF-kappa B signaling pathway	7	9.333333333	1.86E-04	1.86E-04
KEGG_PATHWAY	hsa05226:Gastric cancer	8	10.66666667	1.89E-04	1.89E-04
KEGG_PATHWAY	hsa05130:Pathogenic Escherichia coli infection	9	12	2.07E-04	2.07E-04
KEGG_PATHWAY	hsa05330:Allograft rejection	5	6.666666667	2.29E-04	2.29E-04
KEGG_PATHWAY	hsa05120:Epithelial cell signaling in Helicobacter pylori infection	6	8	2.50E-04	2.50E-04
KEGG_PATHWAY	hsa05223:Non-small cell lung cancer	6	8	2.85E-04	2.85E-04
KEGG_PATHWAY	hsa05170:Human immunodeficiency virus 1 infection	9	12	2.88E-04	2.88E-04
KEGG_PATHWAY	hsa01524:Platinum drug resistance	6	8	3.23E-04	3.23E-04
KEGG_PATHWAY	hsa05020:Prion disease	10	13.33333333	3.56E-04	3.56E-04
KEGG_PATHWAY	hsa05220:Chronic myeloid leukemia	6	8	3.66E-04	3.66E-04
KEGG_PATHWAY	hsa04940:Type I diabetes mellitus	5	6.666666667	3.72E-04	3.72E-04
KEGG_PATHWAY	hsa04630:JAK-STAT signaling pathway	8	10.66666667	3.78E-04	3.78E-04
KEGG_PATHWAY	hsa04140:Autophagy - animal	8	10.66666667	3.92E-04	3.92E-04
KEGG_PATHWAY	hsa04935:Growth hormone synthesis, secretion and action	7	9.333333333	4.21E-04	4.21E-04
KEGG_PATHWAY	hsa04919:Thyroid hormone signaling pathway	7	9.333333333	4.21E-04	4.21E-04

Category	Term	Count	%	PValue	FDR
KEGG_PATHWAY	hsa04930:Type II diabetes mellitus	5	6.666666667	5.25E-04	5.25E-04
KEGG_PATHWAY	hsa04012:ErbB signaling pathway	6	8	6.10E-04	6.10E-04
KEGG_PATHWAY	hsa04672:Intestinal immune network for IgA production	5	6.666666667	6.16E-04	6.16E-04
KEGG_PATHWAY	hsa05131:Shigellosis	9	12	8.13E-04	8.13E-04
KEGG_PATHWAY	hsa04658:Th1 and Th2 cell differentiation	6	8	8.29E-04	8.29E-04
KEGG_PATHWAY	hsa04062:Chemokine signaling pathway	8	10.66666667	8.66E-04	8.66E-04
KEGG_PATHWAY	hsa04912:GnRH signaling pathway	6	8	8.71E-04	8.71E-04
KEGG_PATHWAY	hsa04371:Apelin signaling pathway	7	9.333333333	8.72E-04	8.72E-04
KEGG_PATHWAY	hsa04072:Phospholipase D signaling pathway	7	9.333333333	0.001205514	0.001205514
KEGG_PATHWAY	hsa04150:mTOR signaling pathway	7	9.333333333	0.001629298	0.001629298
KEGG_PATHWAY	hsa04931:Insulin resistance	6	8	0.001773633	0.001773633
KEGG_PATHWAY	hsa05221:Acute myeloid leukemia	5	6.666666667	0.00211259	0.00211259
KEGG_PATHWAY	hsa04024:cAMP signaling pathway	8	10.66666667	0.002155768	0.002155768
KEGG_PATHWAY	hsa05416:Viral myocarditis	5	6.666666667	0.002229143	0.002229143
KEGG_PATHWAY	hsa04928:Parathyroid hormone synthesis, secretion and action	6	8	0.002245205	0.002245205
KEGG_PATHWAY	hsa04622:RIG-I-like receptor signaling pathway	5	6.666666667	0.002605334	0.002605334
KEGG_PATHWAY	hsa04014:Ras signaling pathway	8	10.66666667	0.002883724	0.002883724
KEGG_PATHWAY	hsa05216:Thyroid cancer	4	5.333333333	0.003172358	0.003172358
KEGG_PATHWAY	hsa04611:Platelet activation	6	8	0.003225572	0.003225572
KEGG_PATHWAY	hsa04728:Dopaminergic synapse	6	8	0.004074422	0.004074422
KEGG_PATHWAY	hsa04662:B cell receptor signaling path-way	5	6.666666667	0.006038964	0.006038964
KEGG_PATHWAY	hsa04750:Inflammatory mediator regula-tion of TRP channels	5	6.666666667	0.008108051	0.008108051
KEGG_PATHWAY	hsa05150:Staphylococcus aureus infection	5	6.666666667	0.008395536	0.008395536
KEGG_PATHWAY	hsa04514:Cell adhesion molecules	6	8	0.008418389	0.008418389
KEGG_PATHWAY	hsa04810:Regulation of actin cytoskeleton	7	9.333333333	0.010181217	0.010181217
KEGG_PATHWAY	hsa04914:Progesterone-mediated oocyte maturation	5	6.666666667	0.012007502	0.012007502
KEGG_PATHWAY	hsa04725:Cholinergic synapse	5	6.666666667	0.01353407	0.01353407
KEGG_PATHWAY	hsa04152:AMPK signaling pathway	5	6.666666667	0.016492964	0.016492964
KEGG_PATHWAY	hsa04920:Adipocytokine signaling path-way	4	5.333333333	0.018519073	0.018519073
KEGG_PATHWAY	hsa05012:Parkinson disease	7	9.333333333	0.021313395	0.021313395
KEGG_PATHWAY	hsa04115:p53 signaling pathway	4	5.333333333	0.022206925	0.022206925
KEGG_PATHWAY	hsa01523:Antifolate resistance	3	4	0.023934769	0.023934769
KEGG_PATHWAY	hsa05203:Viral carcinogenesis	6	8	0.024179333	0.024179333
KEGG_PATHWAY	hsa04910:Insulin signaling pathway	5	6.666666667	0.024695783	0.024695783
KEGG_PATHWAY	hsa05322:Systemic lupus erythematosus	5	6.666666667	0.025277209	0.025277209

Category	Term	Count	%	PValue	FDR
KEGG_PATHWAY	hsa05310:Asthma	3	4	0.025454445	0.025454445
KEGG_PATHWAY	hsa04550:Signaling pathways regulating pluripotency of stem cells	5	6.666666667	0.028308518	0.028308518
KEGG_PATHWAY	hsa04623:Cytosolic DNA-sensing pathway	4	5.333333333	0.028882165	0.028882165
KEGG_PATHWAY	hsa04610:Complement and coagulation cascades	4	5.333333333	0.033536627	0.033536627
KEGG_PATHWAY	hsa04540:Gap junction	4	5.333333333	0.037524915	0.037524915
KEGG_PATHWAY	hsa05016:Huntington disease	7	9.333333333	0.038259711	0.038259711
KEGG_PATHWAY	hsa04217:Necroptosis	5	6.666666667	0.038662184	0.038662184
KEGG_PATHWAY	hsa04640:Hematopoietic cell lineage	4	5.333333333	0.045062608	0.045062608
KEGG_PATHWAY	hsa04137:Mitophagy - animal	4	5.333333333	0.052077694	0.052077694
KEGG_PATHWAY	hsa04350:TGF-beta signaling pathway	4	5.333333333	0.055772736	0.055772736
KEGG_PATHWAY	hsa05168:Herpes simplex virus 1 infection	9	12	0.056880249	0.056880249
KEGG_PATHWAY	hsa05030:Cocaine addiction	3	4	0.058725094	0.058725094
KEGG_PATHWAY	hsa05034:Alcoholism	5	6.666666667	0.064099138	0.064099138
KEGG_PATHWAY	hsa04726:Serotonergic synapse	4	5.333333333	0.064867579	0.064867579
KEGG_PATHWAY	hsa05320:Autoimmune thyroid disease	3	4	0.0674312	0.0674312
KEGG_PATHWAY	hsa05014:Amyotrophic lateral sclerosis	7	9.333333333	0.076757681	0.076757681
KEGG_PATHWAY	hsa04923:Regulation of lipolysis in adipocytes	3	4	0.081226273	0.081226273
